# Cross-cultural adaptation and validation of a Brazilian version of an instrument to assess impairments related to oral functioning of people with Down syndrome

**DOI:** 10.1186/1477-7525-11-4

**Published:** 2013-01-11

**Authors:** Karina Bonanato, Isabela A Pordeus, Thiago Compart, Ana Cristina Oliveira, Paul J Allison, Saul M Paiva

**Affiliations:** 1 Department of Pediatric Dentistry and Orthodontics, Faculty of Dentistry, Universidade Federal de Minas Gerais, Av. Antônio Carlos 6627, Belo Horizonte, MG, 31270-901, Brazil; 2 Department of Pediatric Dentistry, Faculty of Dentistry, Universidade Vale do Rio Verde, Rua Gentios 1420, Belo Horizonte, MG, 30380-490, Brazil; 3 Department of Social and Preventive Dentistry, Faculty of Dentistry, Universidade Federal de Minas Gerais, Av. Antônio Carlos 6627, Belo Horizonte, MG, 31270-901, Brazil; 4 Division of Public Health and Society, Faculty of Dentistry, McGill University, 3640 University Street, Montreal, QC, H3A 2B2, Canada

**Keywords:** Down syndrome, Cross-cultural adaptation, Validation, Malocclusion, Oral functioning, Oral health

## Abstract

**Background:**

An instrument was developed in Canada to assess impairments related to oral functioning of individuals with four years of age or older with Down syndrome (DS). The present study attempted to carry out the cross-cultural adaptation and validation of the instrument for the Brazilian Portuguese language and to test its reliability and validity.

**Findings:**

After translation and cross-cultural adaptation, the instrument was tested on caregivers of people with DS. Clinical examination for malocclusion was carried out in people with DS by two calibrated examiners. Inter and Intra examiner agreement was assessed by Intraclass Correlation Coefficient (ICC) and ranged from 0.92 to 0.97 respectively. Total of 157 people with DS and their caregivers were able to compose the sample. They were selected from eight institutions for people with DS in five cities of southeastern Brazil. The mean age of people with DS was 20.7 [±13.1] and for caregivers was 53.1 [±13.7]. The mean instrument score was 18.6 [±9.0]. Internal reliability ranged from 0.49 to 0.80 and external reliability ranged from 0.78 to 0.88. Construct validity was verified by significant correlations identified between malocclusion and the total instrument; and caregivers’ educational level and the instrument (p<0.05). Discriminant validity was proved as the instrument presented different mean comparing people with DS and non-DS (p<0.05).

**Conclusions:**

Initial validity tests indicated that the instrument related to the oral health for people with DS may be a valid instrument to this segment of the population in Brazil.

## Background

The person with Down Syndrome (DS) presents with some special characteristics that may affect the oral functioning [1-3]. The muscular hypotonia and the respiratory infections may increase malocclusion prevalence in this individuals [2,4]. As their motor coordination is affected, the oral hygiene performance is hindered [3,5]. Oliveira et al. [3] developed a study with 112 Brazilian children and adolescents with DS and found 33.0% of the sample exhibited anterior crossbite, 21.0% with anterior open bite and 31.0% with posterior crossbite. Brazil is estimated to have about 300,000 people with DS.

The malocclusion leads to physical and emotional discomfort and may influence negatively the individuals’ quality of life [1-5]. Concerning children and people with intellectual disabilities even their caregivers’ life can be affected [2,6]. The expected limitations of people with DS added with any health problem seems to increase their parents perceived stress and work absenteeism as decrease their perceived health.

Assessment of the oral health in people with intellectual disabilities can be performed by trained professionals using a sort of valid indexes. Most of the oral health indexes are assessed by clinical examination that may be hampered in people with DS because of individuals’ lower capacity of understanding and the staff limitations to lead with special needs [1,3-5,7,8].

Specifically to evaluate the oral health of people with DS, a questionnaire was developed and validated firstly to be applied in Canadian English-speaking caregivers. The instrument assesses some impairment related to oral functioning of people with DS by the report of their caregivers [1,7].

The aim of this study was to perform the translation and cross-cultural adaptation of the oral functioning impairment instrument for people with DS for the Brazilian Portuguese language by examining its psychometric properties and validity.

## Method

### Description of the oral functioning impairment instrument for people with DS

The instrument is specific to people with DS aged from four-years-old or more [7]. The 20 items addressed the frequency of events in the past three months. The items are distributed into four categories of functional impairment: eating, communication, oral parafunction and oral symptoms.

Responses are proposed to be answered in a four-point rating categories. The score is computed by summing all of the categories. Since there were 20 items the final scores can vary from 0 to 60 for which a lower score denotes a better oral health [7].

### Translation and cross cultural adaptation of the instrument

In order to make the instrument comprehensible to the Brazilian population, it was firstly submitted to translation and cross-cultural adaptation [9,10].

Based on standard recommendations, translation was performed by three independent translators to achieve the meaning equivalence. One of them was a native English speaker fluent in Portuguese. The second one was a Brazilian fluent in English. Both of them were English teacher in Brazil. The third translator was a Brazilian dentistry fluent in English. All of them made independent translations.

The semantic equivalence was then performed and this consensus version was discussed with five mothers of people with DS in separate who suggested some word alterations and a format alteration. After this analysis a second synthesis version was obtained.

For the determination of conceptual and item equivalence, a group of experts evaluated this version and compared it to the original. This group consisted of a General clinician, a Psychologist, a Social worker, a Teacher, an Occupational therapist, a Dentist and a Phonoaudiologist. The functional equivalence was obtained from this process. A third synthesis version was developed as a result of this process.

**Figure 1:**
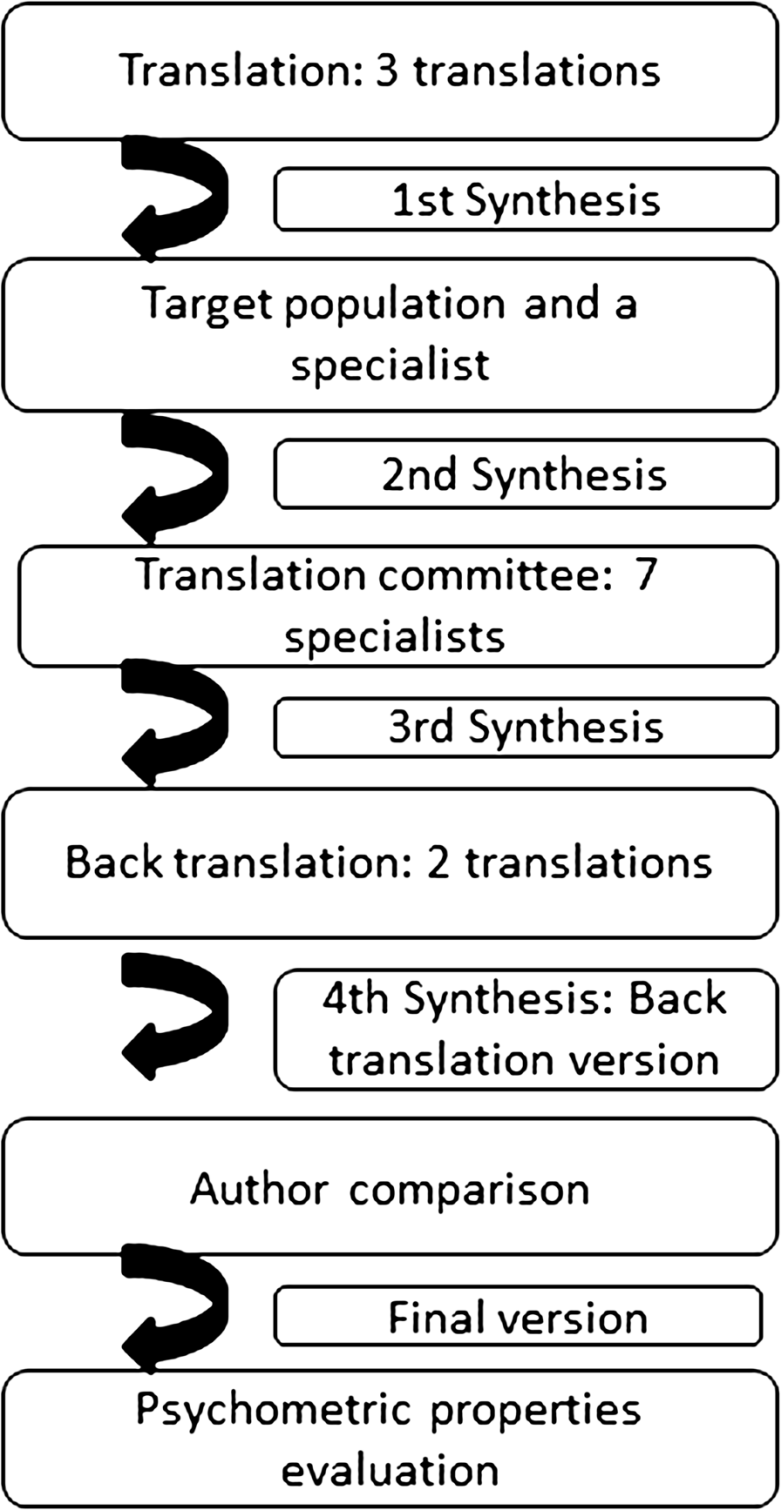
Flow chart of the cross-cultural adaptation steps.

This final version was then translated back into English by two independent native English-speaking translators who were blind to the original English version, as a second step to obtain the content equivalence. These two back-translated English versions were compared and a fourth synthesis version was developed by another Dentist and it was called the Back-translated version. To determine semantic equivalence, the author of the original instrument compared the back-translated version with the original version and found no need for changes. The aim of this step was to achieve a "similar effect" on respondents who speak those different languages (English and Portuguese). The steps of this process are presented in Figure 1. Next the questionnaire was tested for its psychometric properties in the target population.

### Assessment of reliability and validity of the Brazilian version of the instrument

A convenience sample was selected from eight support entities for people with disabilities from five cities in the region of southeastern Brazil. Only people with DS aged from four years or more and their caregivers were included [7].

The study received the approval by the Human Research Ethics Committee of the Federal University of Minas Gerais state.

'Total of 191 participants (people with DS and their caregivers) fulfilled inclusion criterion but 22 pairs of them were excluded to compose a sample for testing clinical calibration. The people with DS were not accompanied by their caregivers were excluded. No patient with a prior history of orthodontic treatment was found. Thus, 169 people with DS and their caregivers were included in the study. Considering the caregivers, 157 of them returned the questionnaire at least once (response rate = 92.3%). Data were collected from this total sample of 157 people with DS, of both sexes, and their caregivers. The caregivers received the questionnaire to be self-completed at home. In the cases when the caregiver was illiterate, the entities provided support staff to help them. The time taken for completion of the instrument was 20 to 30 minutes.

A total of 84 respondents were excluded for test-retest reliability because they could not be contacted in order to answer the questionnaire a second time. Of the total caregivers included, fifty seven completed the questionnaire twice whiting a two-week interval and provided data for the assessment of the test-retest reliability (response rate = 78.1%).

To assess the construct validity of the Brazilian version of the instrument, it was necessary to verify how well the measures the underlying construct being investigated. Thus the oral health functioning impairments questionnaire responses were compared with clinical examination for malocclusion of people with DS and with caregivers’ social condition, assessed by their years of schooling.

Only individuals with DS presenting complete or incomplete permanent denture were included in clinical examination. Those that refused to be examined, who were not present in the institutions at the examination days and who were undergoing orthodontic treatment were excluded. From the 157 people with DS, a total of 82 people fulfilled this eligible criterion and were submitted to clinical examination.

Clinical examination was performed in the institutions. Dental Aesthetics Index (DAI) recommended by the World Health Organization-WHO [11] were used for diagnosing malocclusion. The aesthetic component was not applied in this study. The index can be applied to individuals with complete permanent dentition or with mixed dentition [10,12].

The examiners were two pediatric dentists (KB and TC) that used natural light, disposable mouth mirrors (PRISMA®, São Paulo, SP, Brazil), periodontal probe IPC (WHO-621, Trinity®, Campo Mourão, PE, Brazil), gauze and wooden spatula. The examiners participated of a calibration exercise for malocclusion criteria. Firstly they were trained with photographs and plaster models. In sequence, 22 individuals with DS from one of the entities were examined and re-examined after a two-week interval to assess clinical examination agreement. Inter and Intra examiner agreement for malocclusion was assessed by ICC and ranged from 0.92 to 0.97 respectively.

Analysis of discriminant validity was performed by comparing a group composed of participants with DS and another group consisting of the siblings of the individual with DS who had closest in age and did not presented DS or other alteration. A total of 46 people with DS and their siblings fulfilled eligible criterion. Caregivers completed the questionnaire a second time for controls.

### Data analysis

It was performed by Statistical Package for the Social Sciences software (SPSS for Windows, version 17.0, SPSS Inc, Chicago, IL, USA) adopting a significance level of 5%. The internal consistency was assessed by computing Cronbach’s alpha for the questionnaire and for the categories. Test-retest reliability was tested by ICC.

Correlations between total score and subscales scores with malocclusion and with caregivers’ educational level [13] were verified by Spearman’s correlation coefficient in order to assess the construct validity of the Brazilian version of the instrument.

Paired-*t* test was used to compare the questionnaire answered by the same caregiver for people with DS and their controls in order to assess the discriminant validity.

## Results

People with DS participating in this study were equally distributed by sex, 49.0% males and 51.0% females. Their mean age was 20.7 [±14.1]. Most caregivers were female (95.3%) and their mean age was 53.1 [±13.7]. Regarding education, 50.3% of caregivers had 8 or fewer years of study and 49.7% had more than nine years of study.

The mean scale and subscales scores are presented in Table 1. Malocclusion values varied from 19 to 123. The mean DAI score was 44.2 [±19.3], median 40.6. About 14.0% of them did not present orthodontic treatment need. Other 14.0% presented defined malocclusion, 13.0% severe malocclusion and 57.0% presented very severe or disabling malocclusion.

**Table 1:** Descriptive analysis of the total scale and subscales (n=157)

**Variable**	**Number of Items (score rank)**	**Mean**	**Standard Deviation**	**Median**	**Minimum**	**Maximum**
**Total scale**	20 (0-60)	18.61	9.03	17.00	3	44
**Subscales**						
Eating	4 (0-12)	3.96	3.44	3.00	0	12
Communication	6 (0-18)	6.24	4.23	6.00	0	17
Parafunction	6 (0-18)	5.04	3.08	5.00	0	16
Symptoms	4 (0-12)	3.36	2.52	3.00	0	10

### Reliability

The questionnaire and their subscales achieved an acceptable to good internal consistence and good test-retest reliability. Cronbach’s alpha was 0.80 for the total questionnaire and ranged from 0.50 to 0.80 for the subscales. Test-retest reliability achieved ICC values of 0.88 for the total instrument and ranged from 0.78 to 0.88 for subscales (Table 2).

**Table 2:** Reliability statistics for total scale and subscales

**Variable**	**Number of Items**	**Cronbach alpha(n=157)**	**ICC(95% CI)(n=57)**
**Total scale**	20	0.80	0.88 (0.80-0.93)
**Subscales**			
Eating	4	0.72	0.88 (0.79-0.93)
Communication	6	0.70	0.83 (0.71-0.89)
Parafunction	6	0.49	0.78 (0.64-0.87)
Symptoms	4	0.70	0.83 (0.71-0.90)

### Construct validity

The correlation between malocclusion and the questionnaire as a total score and as the subscales ranged from 0.27 to 0.05. The total score of the questionnaire presented positive and significant correlation with malocclusion. Between the subscales only the “oral symptoms” presented significant correlation with malocclusion. The correlation between caregivers’ educational level and scores of the total questionnaire and scores of subscales showed that only the “eating” and “parafunction” subscales did not have a significant correlation. All of the significant correlations were negative, showing that the best oral health functioning of DS people was correlated with a higher educational level (Table 3).

**Table 3:** Construct validity: rank correlation between total scale and subscales with malocclusion and caregivers’ educational background

**Variable**	**Malocclusion**^**a**^			**Caregivers’ schooling background**^**b**^				
	**r**	P-value*	**r**	P-value*
**Total scale**	0.22	0.04	−0.16	0.05
**Subscales**					
Eating	0.12	0.27	0.01	0.86	
Communication	0.05	0.68	−0.19	0.02	
Parafunction	0.13	0.24	0.03	0.70	
Symptoms	0.27	0.01	−0.24	<0.01	

^a^ n=82 / ^b^ n=151.

**Spearman’s correlation coefficient, significance level of 0.05.

### Discriminant validity

Data addressing discriminant validity is presented in Table 4. It could be observed that there was a significant difference in the scores of the questionnaire and subscales between people with and without DS.

**Table 4:** Discriminant validity: total scale and subscales’ scores for people with DS and their siblings without DS

**Variable**	**DS (n=46)**			**Sibling non DS (n=46)**					
	**Mean (±Standard deviation)**	**Median**	**Mean (±Standard deviation)**	**Median**	P-value*				
Total scale	18.72 (8.48)	17.00	7.63 (5.39)	6.50	<0.01
Subscales					
Eating	3.52 (3.18)	3.00	1.54 (2.35)	1.00	<0.01
Communication	6.22 (4.57)	6.00	1,24 (2.69)	0.00	<0.01
Parafunction	5.33 (3.31)	5.00	2.15 (1.86)	2.00	<0.01
Symptoms	3.65 (2.54)	3.00	2.70 (1.92)	2.00	0.02

*Paired *t*-test, significance level of 0.05.

## Discussion

The oral health functioning impairment instrument for people with Down syndrome is probably the only validated instrument to assess the oral health independently of clinical examination. The Brazilian Portuguese version of the scale exhibited acceptable reliability and validity. The validation and adaptation of instruments related to oral health is important to ensure the comparability and usefulness of research results [7,10,14,15].

To achieve validation it is necessary to follow some standard recommendations. The basic recommendation for this process is in this order: translation, committee approach and back translation [7,10,14-16].

Translation can be performed by two or more professionals. The inclusion of the third professional as fulfilled in this study was designed to assess the clinical importance of the items phrasing that might be of great importance for eventually distinguishing cases in the clinical range [10,16].

The back translation process is important to identify and correct discrepancies that may occur in the translation process. In this study two back translation versions were obtained as suggested by Beaton et al. [15]. The participation of the original instrument’s author increased the assertiveness of the final version in Portuguese language [16].

Questionnaire’s internal consistency was almost equal to those obtained in the original validation which presented values of 0.52-0.79. These results are in fact acceptable as consistency of approximately 0.70 is ideally, over 0.90 it suggests redundancy and under 0.50 it suggests poor internal consistency [17]. Test-retest reliability was considered good and showed similar results with the original study, where it achieved ICC values of 0.64 to 0.84 [7].

### Instrument validity

Construct validity was verified by comparing the instrument scores with clinical examination for malocclusion because it’s a usual abnormality in people with DS [1,3-5]. The disharmony between the bones of the face has a high prevalence in this group [3-5]. Although malocclusions are rarely life threatening, they can cause pain, infection, respiratory complications, and problems with mastication and speech [2-5].

It was expected that parafunction subscale presented association with malocclusion what did not happen [3,4]. In regard of this result, it can be observed that almost all of the six items of parafunctional subscale deal with habits concerning feeding process, which do not have an association with malocclusion. Only the items concerning protruding tongue and grinding teeth could present relationship with malocclusion [3]. The value of 0.49 Cronbach's alpha of the subscale parafunction shows that this is a subscale should not be used alone. This way maybe the parafunction subscale needs to be correlated with other clinical measures to assure its validity. That only makes sense if used in the complete instrument, which showed a value of 0.80, being quite acceptable.

It was expected that communication subscale was correlated with malocclusion as the deformities in the overall oral cavity lead DS people to present problems in their speech development. Due to malformation of the nasal bones, muscle hypotonia and the large tongue, the DS people usually keeps his mouth open and the tongue between his lips [3,4]. Regarding this results it can be observed that half of the six items concerning communication subscale deal with speech capability.

Eating capability of DS people is in fact affected by the reduced production of saliva, the large tongue, the small oral cavity, oral hypotonia, abnormal tongue movement and uncoordinated sucking and swallowing. Besides, other overall health problems will affect their eating capacity, as digestive dismotility [3,4]. Thus the eating subscale may be influenced by the overall health more than by the malocclusion severity.

Symptoms subscale presented a significant correlation with malocclusion although the index is based on aesthetic features. It must be noticed that items of this subscale concerns about pain, bleeding gums, bead breath and the role perception about the oral health. This way, symptoms subscale was in fact expected to present correlation with malocclusion, instead its’ aesthetical feature.

Otherwise, there was a significant correlation between the instrument total assumed as a scale with malocclusion, proving the construct validity despite the results for the subscales in separate.

In the original validation study for the English version, all of the subscales presented no correlation with the clinical outcomes observed, caries experience and periodontal status. The instrument assumed as a role scale was not assessed in the Canadian study [7]. This suggests that subscales may not present construct validity in separate, but only when analyzed as a single measure.

Educational level was selected to be a second variable aiming to verify the construct validity as social support influences the overall health of people more than economic status [13]. The current study considered years of schooling (caregivers) as proxies for socioeconomic status in the assessment of independent negative impacts in the instrument. Low educational level may leads to reduced income, unemployment and poor occupational status. These conditions influence the health behaviours and self-rated oral health [1,3,4,10]. The educational level did not presented association with people with DS oral health indexes in another study [5]. Results pointed out that caregivers’ schooling was correlated with the scale and the subscales communication and symptoms. It is possible that those aspects are in fact correlated with educational level [3,4,6]. In conclusion, the construct validity of the overall scale seems to be proved but for subscales it must be observed with careful.

Probably the weak correlations occurred because the instrument has very subjective questions and also for being a proxy measure, which not always represents the real oral health status of the individual with Down syndrome. Moreover, measures with fewer items tend to be more sensitive to this analysis. The authors of the original instrument have not found high values in the respective correlations [7].

Discriminant validity proved to be valid. The results proved that the instrument as a scale and the subscales behaves differently within DS and non-DS individuals. As in the original study, the Brazilian version of the scale discriminates the specifically problems of DS people and the result was similar [7].

There are several limitations in this study that should be pointed out. First the sample selection could arise some doubts. Convenience sample increases the possibility of bias and may lead the sample to be similar in many aspects. The DS people in the role population may present different characteristics. There was a greater range of groups’ age. Thus the caregivers of the oldest people with DS were not their parents but other people closest in age, as their brothers. They may not notice about their health conditions or may have a worst expectancy compared with the youngest caregivers as the DS overall heath seems to be improving as their life expectancy [2,3]. Other limitation of this study is that the questionnaire is a proxy measure, which not always represents the real oral health condition [8].

The oral health conditions affect quality of life of all types of individuals. Thus, check symptoms, functional limitations, emotional and social wellbeing related to oral cavity should be considered when evaluating the patient and the population overall health [6,8,10]. This scale has an important role in the evaluation of oral conditions of individuals with DS, identifying, through the report of the caregivers, the impact of oral diseases, mainly of malocclusion on quality of life in this part of the population.

## Conclusion

Initial validity tests indicated that the instrument related to the oral health for people with DS may be a valid instrument to this segment of the population in Brazil.

## Abbreviations

DAI: Dental Aesthetics Index; DS: Down syndrome; ICC: Intraclass Correlation Coefficient; SPSS: Statistical Package for the Social Sciences; WHO: World Health Organization.

## Competing interests

The authors declare that they have no competing interests.

## Authors’ contributions

KB, IP, TC, AO, PA and SP conceptualized the rationale and designed the study. KB and TC performed the data collection. KB and IP performed the statistical analysis and interpretation of the data. KB, AO, SP and PA conducted the literature review and drafted the manuscript. All authors read and approved the final manuscript.
